# Morphology, Photocatalytic and Antimicrobial Properties of TiO_2_ Modified with Mono- and Bimetallic Copper, Platinum and Silver Nanoparticles

**DOI:** 10.3390/nano9081129

**Published:** 2019-08-06

**Authors:** Izabela Wysocka, Ewa Kowalska, Jacek Ryl, Grzegorz Nowaczyk, Anna Zielińska-Jurek

**Affiliations:** 1 Department of Process Engineering and Chemical Technology, Faculty of Chemistry, Gdańsk University of Technology, G. Narutowicza 11/12, 80-233 Gdańsk, Poland; 2 Institute for Catalysis (ICAT), Hokkaido University, N21, W10, Sapporo 001-0021, Japan; 3 Department of Electrochemistry, Corrosion and Materials Engineering, Faculty of Chemistry, Gdańsk University of Technology, G. Narutowicza 11/12, 80-233 Gdańsk, Poland; 4 NanoBioMedical Center, Adam Mickiewicz University, Umultowska 85, 61-614 Poznan, Poland

**Keywords:** photocatalysis, noble metals, nanoparticles, titanium (IV) oxide, photooxidation, hydrogen generation

## Abstract

Noble metal nanoparticles (NMNPs) enhanced TiO_2_ response and extended its activity under visible light. Photocatalytic activity of TiO_2_ modified with noble metal nanoparticles strongly depends on the physicochemical properties of NMNPs. Among others, the differences in the size of NMNPs seems to be one of the most important factors. In this view, the effect of the metal’s nanoparticles size, type and amount on TiO_2_ photocatalytic and biocidal activity was investigated. TiO_2_ modified with mono- and bimetallic nanoparticles of Pt, Cu and Ag were prepared using chemical and thermal reduction methods. Obtained nanocomposites were characterized using transmission electron microscopy (TEM), X-ray photoelectron spectroscopy (XPS), X-ray diffraction (XRD) and diffuse-reflectance spectroscopy (DR/UV-Vis) techniques. The photocatalytic activity was examined in 2-propanol oxidation and hydrogen generation processes. The mechanism of modified TiO_2_ excitation was evaluated in action spectrum measurements during phenol oxidation. A possibility of using less energy-consuming light sources as a set of light-emitting diodes (LEDs) selected based on action spectrum results was examined. It was found that the differences in NMNPs size were the result of the reduction method. Moreover, coupling with a second metal strongly affected and differentiated the photocatalytic and biocidal activity of the obtained TiO_2_-based photocatalysts.

## 1. Introduction

Noble metal nanoparticles (NMNPs) have attracted much attention due to their unique properties in comparison to bulk metals. They possess exceptional optical, electrical and magnetic properties and are therefore applied in various branches of industry, such as electronics, pharmacy, catalysis, cosmetics, optoelectronics and medicine [1,2,3].

Metal nanoparticles of *d* block (Ag, Au, Cu, Pt and Pd) possess the ability to absorb light from the visible to the near-infrared range due to the occurrence of localized surface plasmon resonance (LSPR). The term localized surface plasmon resonance (LSPR) describes the oscillation of metal particle-free electrons. The irradiation of nanoparticles with radiation of resonant frequency results in the oscillations of free electrons. If the frequency of the exciting wavelength is the same as the particle oscillation frequency, the particle is set into oscillatory vibrations. Alternating oscillating charges of the resulting dipole emits electromagnetic waves of different frequencies. Since LSPR is an excited state, the return of a particle to the ground state proceeds via either a radiative or non-radiative way. Radiation suppression consists of photon emission, while non-radiative suppression consists of the oscillation of each electron with a charge transfer. The electron can be transferred as either interband or intraband, initiating local changes in the electric field in the surrounding environment [4,5,6,7,8,9,10,11,12,13].

The phenomenon of LSPR found an application in heterogeneous photocatalysis. The photocatalytic reaction consists in the excitation of a semiconductor with energy equal to or greater than its bandgap energy leading to the generation of charge carriers (electron–hole pair) and further formation of reactive oxygen species (ROS). Among examined semiconductors, the most widely used is titanium(IV) oxide, mainly due to its high photocatalytic activity, low cost, chemical and thermal stability [14,15]. Its photocatalytic properties have already been studied in water splitting, CO_2_ conversion to hydrocarbons, self-cleaning surfaces, water and air purification [16,17]. However, due to the large bandgap energy, it can be excited only by UV irradiation (λ < 388 nm), which requires the application of high-energy consuming UV lamps as an irradiation source and limits utilization of green light sources, like solar light. In order to spread TiO_2_ optical response over the visible light region, many methods of surface modification or doping of the semiconductor structure have been studied [18,19,20,21]. Among them, surface modification with noble and semi-noble metal (Ag, Au, Pt, Pd, Cu) nanoparticles was proposed. At the metal-semiconductor interface upon UV light irradiation, a Schottky barrier is created, which retards the charge carriers recombination by trapping electrons. Under visible light irradiation, TiO_2_ is activated during LSPR decay through electron transfer from metal nanoparticles (Ag, Au, Cu, Pt or Pd) to titanium(IV) oxide conduction band [5,9,22,23,24,25].

Apart from plasmonic properties, silver or copper nanoparticles deposited on TiO_2_ induces biocidal properties of the obtained photocatalyst [26,27,28]. Antimicrobial properties may result from the ability of the photocatalyst to generate reactive oxygen species and selected features of modifiers. Silver and copper nanoparticles are able to inhibit bacteria growth cells through binding with cysteine thiol groups leading to protein inactivation [3,29,30]. Generation of ROS leads to structural changes and the lysis of bacteria cell-walls by the peroxidation of organic components [28].

The type, size and shape of deposited metals may affect photocatalytic activity and selectivity of TiO_2_-based nanocomposites [4,12,21,31,32]. It was reported that, depending on the reducing agent, an oxygen saturation, the titanium(IV) oxide crystalline phase and calcination temperature, metal nanoparticles (NPs) of various size, shape, oxidation state and distribution are formed [12,19,26,31,33,34]. Dong et al. [32] investigated the effect of platinum nanoparticles size on activity and selectivity during carbon(IV) oxide photocatalytic reduction. It was found that a decrease of the size of platinum particles enhances the charge transfer efficiency leading to more effective hydrogen generation and CO_2_ photocatalytic reduction. However, the photocatalytic activity of TiO_2_ nanocomposites depends not only on metal NPs properties but also on properties of titania such as size, crystalline structure, specific surface area, surface hydroxylation and surface functionalization with non-metal and metal elements. In our previous study, we reported that photocatalytic activity strictly depended on the type and size of Pt, Pd, Cu and Au nanoparticles as well as TiO_2_ morphology [26,35]. Anpo et al. [36] investigated the photocatalytic activity of various commercial TiO_2_ JCR-TIO under UV light. The examined photocatalysts differed from the crystal structure, specific surface area, bandgap energy and a number of hydroxyl groups. They found that the most active titania was characterized by anatase structure, the large bandgap and the high concentration of surface hydroxyl groups. Murcia et al. [37] investigated the effect of surface functionalization of TiO_2_ with fluorine and sulfate ions combined with platinum nanoparticles deposition. They found that Pt NPs significantly enhanced the photocatalytic activity of sulfated TiO_2_, while it had a minor effect on the activity of fluorine titania. Thus, defining the correlation between structural properties and photocatalytic activity, as well as selectivity and biocidal properties should be of particular interest, especially when designing high-efficiency and environmentally-friendly photocatalytic processes.

In this regard, the effect of metal nanoparticles type, size, and content on photocatalytic and biocidal activity was investigated. Nanocomposites of TiO_2_ modified with mono-/bimetallic nanoparticles of platinum, copper and silver were obtained via chemical and thermal reduction methods. For the first time, the photocatalytic activity and properties of bimetallic TiO_2_-based photocatalysts in reduction and oxidation processes were studied. The spectral activity was analyzed during the phenol oxidation reaction under monochromatic irradiation in the range of 320 to 570 nm. Based on action spectrum results, a set of light-emitting diodes (LED) was selected and used as an irradiation source for the photocatalytic oxidation of organic pollutants. A biocidal activity was investigated against an *Escherichia coli* strain.

## 2. Materials and Methods

### 2.1. Materials

Commercial titanium (IV) oxide ST-01 (supplier: Ishihara Sangayo Ltd., Osaka, Japan) was used as TiO_2_ matrix. Silver nitrate, copper nitrate, potassium tetrachloroplatinate were purchased from Sigma Aldrich (Sigma Aldrich, Saint. Louis, MO, USA) and used as a starting material for the metals nanoparticles preparation. Sodium borohydride was supplied by POCh S.A. (Gliwice, Poland) and used as a reducing agent. 2-propanol and phenol, selected as model pollutants, were obtained from Fluka (Shanghai, China). Methanol was purchased from Fluka (Shanghai, China) and selected as a sacrificial agent during the hydrogen generation reaction.

### 2.2. Characterization Techniques

Diffuse-reflectance spectra (DR/UV-Vis) were recorded in the range of 300 to 800 nm and converted to absorption spectra. Bandgap energies were calculated from the corresponding Kubelka–Munk function F(R), which is proportional to the absorption of radiation, by plotting F(R) 0.5 E_ph_^0.5^ against E_ph_, where E_ph_ is photon energy. The measurements were carried out using ThemoScientific Evolution 220 Spectrophotometer (Waltham, MA, USA) equipped with a PIN-757 (Waltham, MA, USA) integrating sphere. As reference samples, commercial TiO_2_ ST-01 and BaSO_4_ were used.

X-ray diffraction (XRD) analysis was performed using Rigaku Intelligent X-ray diffraction system SmartLab equipped with a sealed tube X-ray generator (Rigaku Corporation, Tokyo, Japan) (a copper target; operated at 40 kV and 30 mA), a D/teX high-speed position sensitive detector system and an ASC-10 automatic sample changer. Data acquisition conditions were as follows; 2θ range: 20°–90°, scan speed: 1°·min^−1^ and scan step 0.008. The obtained XRD patterns were analyzed by Rigaku PDXL (Version 2.0, Rigaku Corporation, Neu-Isenburg, Germany, 2007), a crystal structure analysis package including Rietveld analysis, installed in a computer controlling the diffractometer.

The surface composition was analyzed using X-ray photoelectron spectrophotometer (XPS) equipped with EDS detector. Powdered samples were attached to conductive carbon tape and a copper holder for XPS analysis and dried overnight under vacuum. All XPS spectra were recorded on Escalab 250Xi (Walthman, MA, USA), Thermofisher Scientific spectrometer using Mg K X-rays.

The morphology, size, and distribution of nanoparticles were investigated using HR-TEM HRTEM Jeol ARM 200F microscope (Jeol-USA, Peabody, MA, USA) operated at 200 kV and Cs-corrected STEM (High Angle Annular DarkField—HAADF) or high-resolution transmission electron microscope (HRTEM) Tecnai F20 X-Twin (Fei Company, Hillsboro, OR, USA).

### 2.3. Preparation of Photocatalysts

Two series of metal-modified titanium(IV) oxide photocatalysts were prepared. In the first series, metal ions were chemically reduced on the TiO_2_ surface using sodium borohydride, while in the second series metal ions were reduced thermally.

Firstly, 2 g of commercial titanium(IV) oxide (ST-01) was dispersed in 50 cm^3^ of water. Next, suitable volumes of 0.1 M metal salts (K_2_PtCl_4_, AgNO_3_ or Cu (NO_3_)_2_) solutions were added dropwise. Metal contents used for preparation ranged from 0.05 to 0.5 mol. % TiO_2_ (see in Table 1). Obtained dispersions have been further mixed to provide equal adsorption of metal ions on TiO_2_ surface. For samples reduced chemically, metal ions were reduced by the dropping of 0.1 M aqueous solution of sodium borohydride in molar excess equal to 1.5. Obtained metal-modified TiO_2_ powders were centrifuged, dried at 80 °C to dry mass and calcined at 400 °C for 2 h. The rate of heating during the calcination process was maintained at 2 °C·min^−1^.

### 2.4. Photocatalytic Activity

#### 2.4.1. 2-Propanol Photocatalytic Oxidation

A total of 50 mg of the photocatalyst was dispersed in 5 cm^3^ of 2-propanol solution (5 vol. %). The obtained suspension was irradiated with a 300 W Xenon lamp (Hamamatsu Photonics, Hamamatsu, Japan). Measurements were conducted under UV-vis (λ > 400 nm) and vis light (λ > 450 nm). Irradiation of wavelengths shorter than 450 nm were cut-off using a Y48 filter (Hamamatsu Photonics, Hamamatsu, Japan). The photocatalytic activity was evaluated as a function of acetone generation as acetone is the first intermediate product of 2-propanol photocatalytic mineralization to carbon (IV) oxide. Acetone concentration was determined chromatographically using a Shimadzu GC-8A chromatograph equipped with PEG-20M Unipart B (Agilent Technologies, Santa Clara, CA, USA) column and FID detector (Shimadzu Corporation, Kyoto, Japan). The amount of generated CO_2_ during the reaction was measured using Shimadzu GC-14B with a flame-ionization detector (FID) and methanizer (Shimadzu Corporation, Kyoto, Japan).

#### 2.4.2. Hydrogen Generation

A total of 50 mg of the photocatalyst was suspended in 5 cm^3^ of 50 vol. % of the aqueous methanol solution. The obtained suspension was purged with argon in order to remove dissolved oxygen. The concentration of residual oxygen was measured chromatographically with a Shimadzu GC-8A Chromatograph (column MS-5A ((Agilent Technologies, Santa Clara, CA, USA), thermal conductivity detector (TCD)) (Shimadzu Corporation, Kyoto, Japan). After the complete removal of oxygen from the reaction solution, the suspension was irradiated with a 400 W mercury lamp (Hamamatsu Photonics, Hamamatsu, Japan). Generated hydrogen was determined chromatographically using the same set-up as described for oxygen determination.

#### 2.4.3. The Spectral Activity of Phenol Photocatalytic Oxidation

The apparent quantum yield of phenol oxidation was quantified at wavelengths 320, 380, 440, 450, 510 and 570 nm. A photocatalyst at the content of 2 G·dm^−3^ was added to the aqueous phenol solution (c = 20 mg·dm^−3^) and kept for 30 min in darkness to provide equilibrium conditions. The obtained suspension was irradiated with monochromatic light at the specific intensity in the range of 2–4 mWcm^−2^ emitted by a diffraction grating type illuminator (Jasco CRM-FD (Jasco Corporation, Tokyo, Japan)) equipped with a 300 W xenon lamp (Hamamatsu Photonics, Hamamatsu, Japan). The intensity of the radiation was measured using a Hioki 3664 Optical Power Meter (Hioki E.E. Corporation, Nagano, Japan). The apparent quantum yield of the phenol oxidation process was determined as a function of the 1,4-benzoquinone generation as the first photocatalytic oxidation intermediate product. The concentration of 1,4-benzoquinone was determined using a high-performance liquid chromatograph Shimadzu LC-6A ((Shimadzu Corporation, Kyoto, Japan) equipped with a WAKOSIL-II 5C18 AR column (4.6 × 250 mm) (FUJIFILM Wako Pure Chemical Corporation, Osaka, Japan) and a UV-Vis Shimadzu SPD-6A detector (Shimadzu Corporation, Kyoto, Japan)., detection wavelength at 254 nm. The mobile phase consists of water, acetonitrile and phosphoric acid (V) solution in a volume ratio 70:29.5:0.5, respectively. The mobile phase flow was maintained at 0.5 cm·dm^−3^.

#### 2.4.4. Photocatalytic Phenol Oxidation in UV/Vis-LED System

A total of 1.4 g of the photocatalyst was suspended in 700 cm^3^ of phenol solution (C_0_ = 20 mg·dm^−3^) and mixed in darkness in order to establish equilibrium. The light source consisted of a set of light-emitting diodes (LED) covered with borosilicate glass dipped in the reaction suspension. The diodes emitted radiation in the UV-Vis range λ = 380–440 nm with the emission maximum at λ_max_ = 415 nm. The radiation flux was maintained at 2.5 mW·cm^−2^ at 415 nm and controlled using Hioki 3664 m with the Hioki 9742 sensor (Hioki E.E. Corporation, Nagano, Japan). The emission spectrum of diodes used is shown in Figure S1.

#### 2.4.5. Biocidal Properties

Biocidal activity in the aqueous phase was tested against *E. coli (K12)* strain. The analysis of biocidal activity was performed under visible light irradiation (λ > 450 nm) and in the dark. In this regard, 50 mg of the photocatalyst was dispersed in a test tube filled with 7 cm^3^ of bacterial cell suspension in physiological saline (0.9% NaCl). The optical density of the cell suspension ranged from 0.5 to 1 according to McFarland standards, which corresponds to a cell concentration of approximately 1.5 × 10^8^ CFU·cm^−3^ (colony-forming unit/cm^3^). A content of viable cells was determined using serial dilution method on Petri plates filled with PCA agar (Plate Count Agar Becton, Dickinson and Company, Sparks, MD, USA). The plates were incubated for 24 h at temperature 310 K.

## 3. Results

### 3.1. Characterization of Nanocomposites

Sample labeling, metal contents used for preparation, the average crystallite anatase size and reaction rate constants are presented in Table 1. Letters C and T refer to nanocomposites obtained by chemical and thermal reduction of metal ions, respectively.

Diffraction patterns of metal-modified TiO_2_ are shown in Figure S2a–c All obtained photocatalysts revealed a high level of crystallinity and consist of an anatase phase. Reflections observed at 2θ of 25.28°; 37.89°; 47.84°; 54.59°; 62.49°; 69.68°; 75.10° and 82.63° correspond to anatase crystal planes (1 0 1), (0 0 4), (2 0 0), (1 0 5), (2 0 4), (1 1 6), (2 1 5) and (0 0 7), respectively [38,39]. The average anatase crystallite size ranged from 12 nm to 14 nm. Only for Ag/Pt-TiO_2__C and Ag/Pt-TiO_2__T diffraction peaks attributed to metal nanoparticles (NPs) were observed (see Figure S2c). Reflections at 2θ = 32.1° and 46.1° observed for Ag/Pt-TiO_2__C correspond to silver(II) oxide and platinum, respectively [26,40]. For the photocatalyst Ag/Pt-TiO_2__T, the average crystallite size calculated based on the Scherrer formula was equal to 57 nm for AgO and 24 nm for Pt, while for Ag/Pt-TiO_2__C the crystallite size for AgO and Pt was 27 nm and 34 nm, respectively. It was observed that the method of metal ions reduction strongly affects the size of metals crystallites. Thermal reduction of silver ions resulted in a crystallite size that was two times larger, while for platinum ions thermal reduction led to a reduction of crystallite size from 34 nm to 24 nm. The metal-TiO_2_ binding energies determine the rate of metal diffusion on the surface of titanium (IV) oxide and the growth of metal clusters. The higher the binding energy, the smaller the diffusion rate of deposited metal and smaller metal clusters were deposited on the TiO_2_ surface. Binding energy (−6.05 eV) of platinum and TiO_2_ in the oxidized form are significantly higher compared to the binding energy (−2.28 eV) of Pt and TiO_2_ in the reduced form. Therefore, the formation of smaller Pt species was observed using the thermal reduction method. For silver, the growth of crystallites results mainly from high-temperature annealing [41,42]. Therefore, chemical reduction using a strong reducing agent prevents crystallite growth and leads to the production of smaller silver particles.

**Figure 1:**
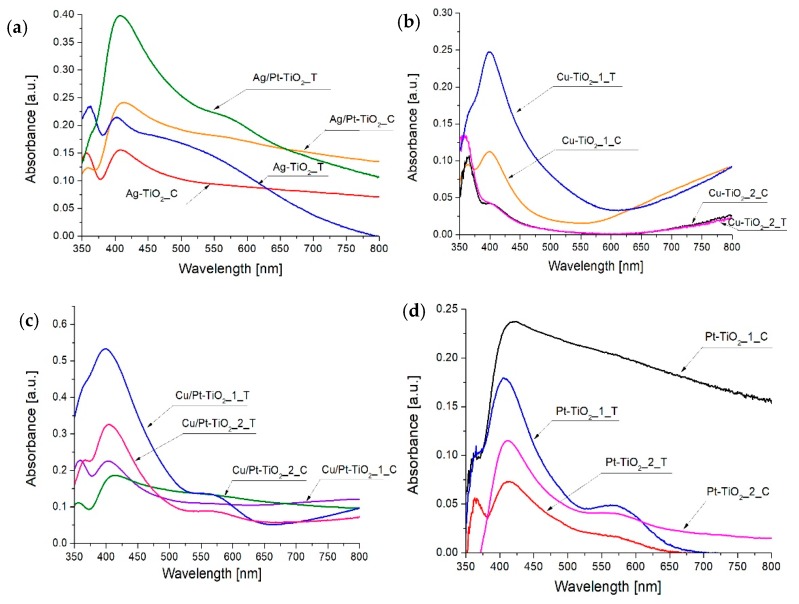
Absorption spectra of (**a**) Ag-TiO_2__C, Ag-TiO_2__T, Ag/Pt-TiO_2__T and Ag/Pt-TiO_2__C (**b**) Cu-TiO_2__1_C, Cu-TiO_2__2_C, Cu-TiO_2__1_T and Cu-TiO_2__2_T, (c) Cu/Pt-TiO_2__1_C, Cu/Pt-TiO_2__2_C, Cu/Pt-TiO_2__1_T and Cu/Pt-TiO_2__2_T, (**d**) Pt-TiO_2__1_C, Pt-TiO_2__2_C, Pt-TiO_2__1_T and Pt-TiO_2__2_T.

Absorption spectra of thermally and chemically reduced photocatalysts are presented in Figure 1a–d. As the reference material, unmodified TiO_2_ was used. All metal-modified photocatalysts revealed an enhanced absorption of visible light in comparison to bare TiO_2_. Three main absorption bands: In the range of 400–450 nm, 550–600 nm and 700–800 nm, were observed for metal-modified TiO_2_ composites. An increase in absorption in the range from 400 to 450 nm is attributed to the surface plasmon resonance phenomenon of platinum and silver nanoparticles [43,44]. For photocatalysts modified with copper nanoparticles, an increase in absorption intensity in the range from 400 to 450 nm corresponds to electron transport between the TiO_2_ valence band and Cu(II) [45]. Absorption region from 550 to 580 nm is attributed to surface plasmon resonance of copper nanoparticles [46,47] and to the electron transport between Pt(IV) and Pt(II) species [26]. The last absorption band observed for copper-modified photocatalysts in the range from 650 to 800 nm indicates the presence of copper ions Cu(II) and Cu(I) [19,45,48]. It was observed that the metal ions reduction method strongly influences the shape of absorption properties. For chemically reduced photocatalysts, absorption bands were wide and fuzzy over the entire spectrum, whereas for thermally reduced photocatalysts narrower absorption bands were observed, as shown in Figure 1a–d. Differences in the shape of the spectra of individual metals deposited on TiO_2_ may result from the difference in the distribution of particle sizes [26,49]. The bandgap energy for all photocatalysts were equal to 3.2 eV, indicating surface modification of TiO_2_ with metal nanoparticles.

**Figure 2:**
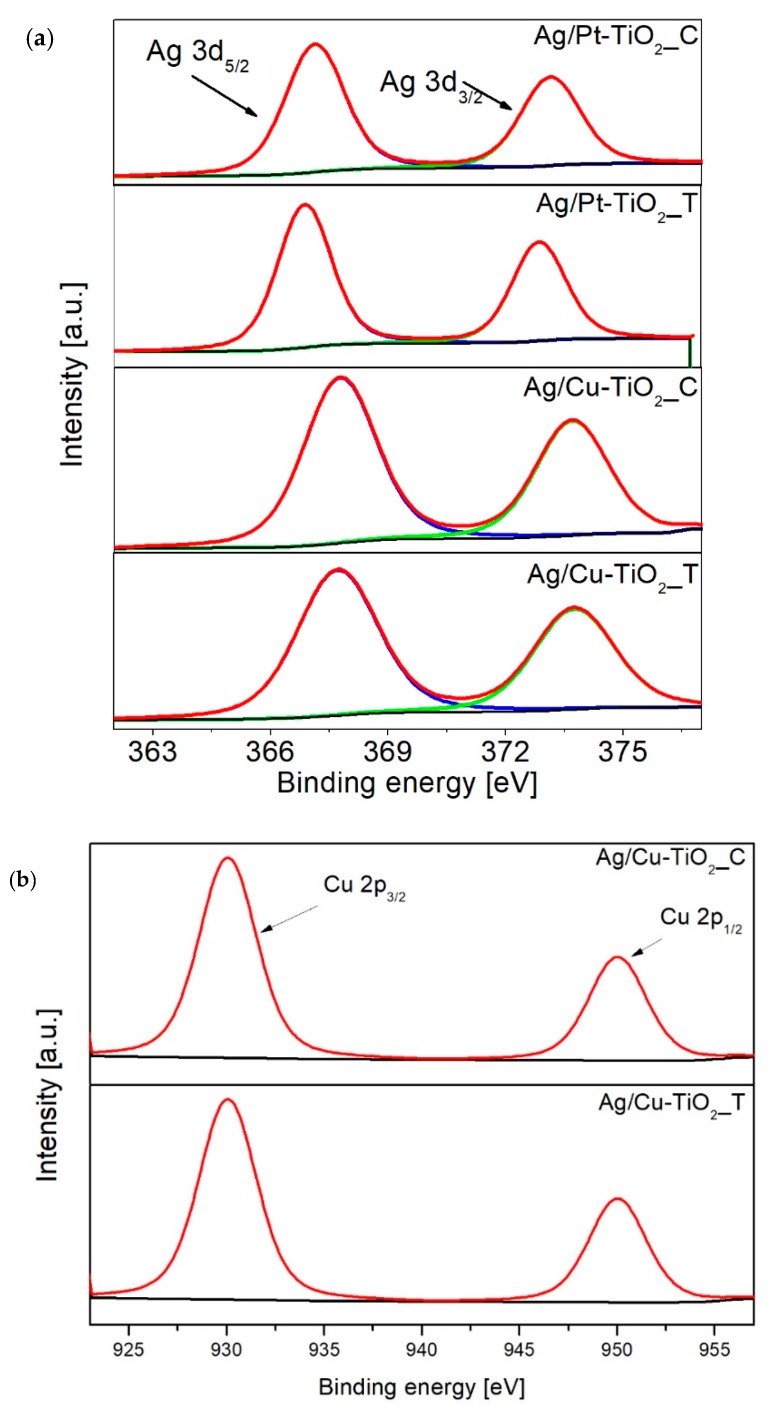

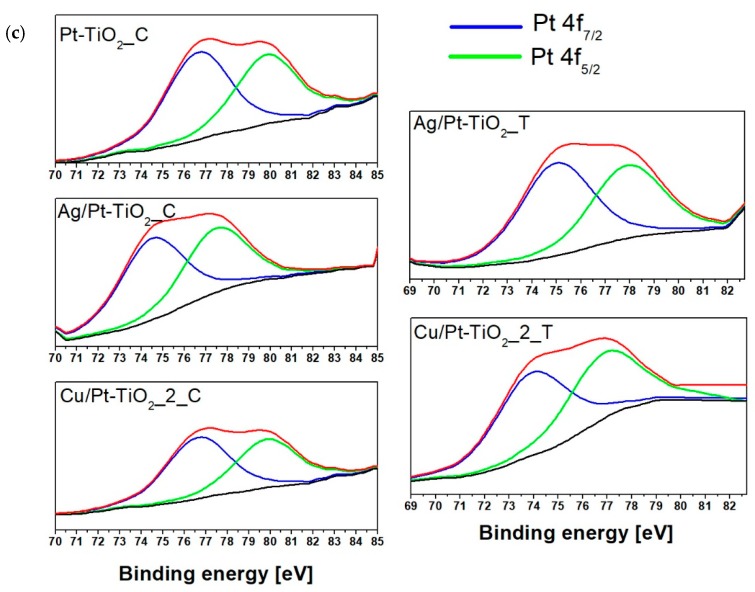
Deconvolution of X-ray photoelectron spectroscopy (XPS) spectra for (**a**) Ag 3d, (**b**) Cu 2p and (**c**) Pt 4f.

Results of surface composition analysis using X-ray photoelectron spectroscopy are presented in Table 2 and Figure 2a–c. All examined photocatalysts revealed similar element composition. The titanium content ranged from 23 at. % to 27 at. %. For each photocatalyst, the signal attributed to the Ti 2p region was deconvoluted into two-component peaks at binding energy (BE) 458.6 eV and 457.2 eV, which refer to Ti^4+^ and Ti^3+^ states, respectively [33,50,51]. The oxygen content ranged from 59.4 to 62.9 atomic percent. As a result of O 1s peak deconvolution, two-component peaks at 530 eV and 531 eV attributed to Ti–O groups and Ti–OH surface hydroxyl groups [50] were observed. Carbonaceous deposits observed on TiO_2_ surface ranged from 9.9 to 13.9 at. %. During the preparation of TiO_2_ photocatalysts, no organic compounds that could be a source of carbon were used, therefore the origin of present carbon is attributed to atmospheric contaminants. The obtained signal for carbon was divided into three component peaks at binding energy 284.8 eV, 285.2 eV and 289.0 eV, which refer to C–C, C–H and C=O bonds [52], respectively. The content of metal nanoparticles ranged from 0.1 at. % to 0.4 at. %. The surface composition analysis revealed differences in the chemical state of silver nanoparticles between chemically and thermally reduced photocatalysts. For Ag/Pt-TiO_2__C and Ag/Cu-TiO_2__C nanocomposites, silver appeared in the form of Ag^0^ and Ag^2+^. The Ag 3d_5/2_ peak at 367.3 eV and Ag 3d_3/2_ at 373.1 eV corresponded to silver Ag^2+^ in the form of oxide and Ag^0^ [43]. For both photocatalysts, the dominant fraction was silver Ag^2+^. For the nanocomposites modified with silver and platinum, as well as copper and silver obtained using thermal reduction, the presence of Ag^0^ and Ag^+^ states was also observed, in which the dominant fraction was silver(I) oxide. For Ag/Pt-TiO_2__T and Ag/Cu-TiO_2__T peaks were attributed to Ag 3d_5/2_ (BE = 367.7 eV) and Ag 3d_3/2_ (BE = 373.7 eV) corresponding to Ag_2_O and Ag^0^ were identified [43].

The presence of platinum species was confirmed by deconvolution of Pt 4f peak into two components Pt 4f_7/2_ and Pt 4f_5/2_. Observed signals at binding energies of 75.0, 74.8 and 74.2 eV referred to Pt^0^ and signals at 77.5 and 77.9 eV referred to Pt^4+^. Peaks attributed to Pt 4f region were characterized by a comparable surface irrespective of the reduction method. For Ag/Cu-TiO_2__T and Ag/Cu-TiO_2__C, the region referring to Cu 1s peaks at binding energies of 930.1 eV and 950.0 eV were observed [53]. For Cu/Pt-TiO_2__2_T and Cu/Pt-TiO_2__2_C, peaks attributed to copper species were not observed due to the low content of the deposited metal.

**Figure 3:**
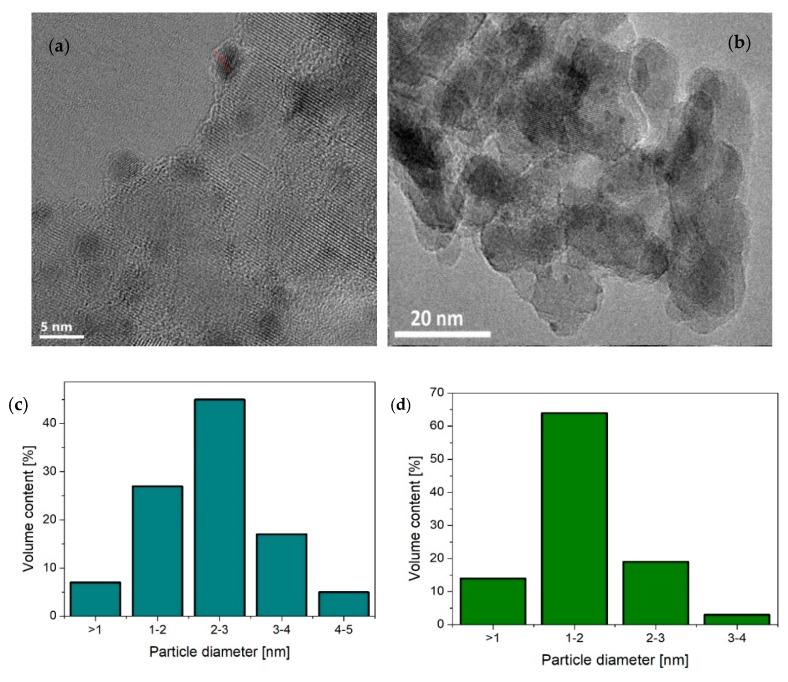

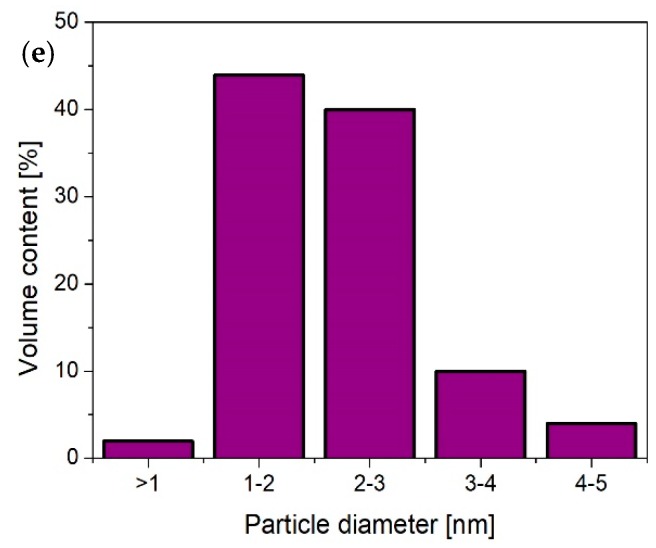
Transmission electron microscopy (TEM) images of (**a**) Pt-TiO_2__2_C, (**b**) Pt-TiO_2__2_T and metal particles size distribution for (**c**) Pt-TiO_2__2_C, (**d**) Pt-TiO_2__2_T and (**e**) Cu/Pt-TiO_2__2_C.

**Figure 4:**
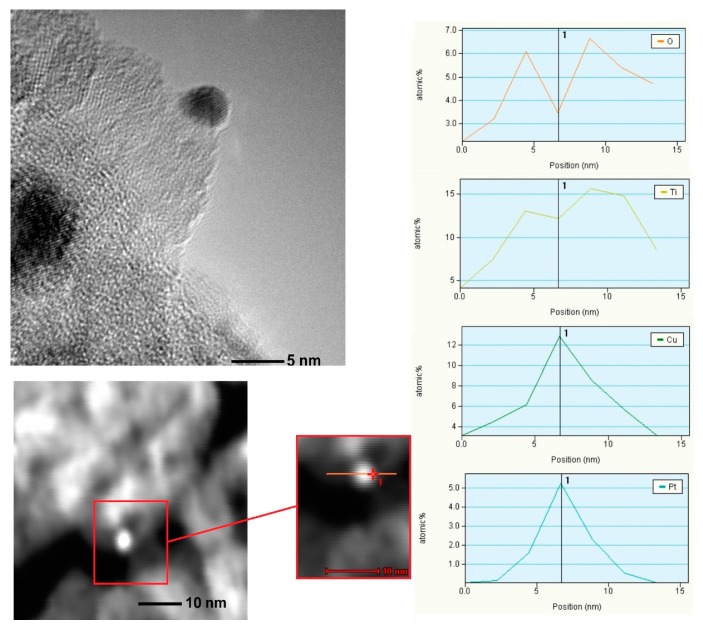
TEM images and line analysis of Cu/Pt-TiO_2__2_C.

Transmission electron microscopy (TEM) images of Pt-TiO_2__2_C, Pt-TiO_2__2_T, Cu/Pt-TiO_2__2_C, Ag/Pt-TiO_2__T and Ag/Pt-TiO_2__C are presented in Figure 3a–e, Figure 4 and Figures S3 and S4 in the Supplementary Materials. The analysis confirmed the surface modification of titanium(IV) oxide with platinum, silver and copper species. For Pt-TiO_2__2_C, Pt-TiO_2__2_T and Cu/Pt-TiO_2__2_C photocatalysts, metal nanoparticles revealed the spherical shape and uniform distribution on TiO_2_. The size of Pt particles in the Pt-TiO_2__2_C nanocomposite ranged from 1 nm to 6 nm, and the average particle size was equal to 3.4 ± 0.9 nm. The main fraction consists of nanoparticles with sizes ranging from 3 nm to 4 nm. Platinum nanoparticles obtained as a result of thermal reduction were twice as small in comparison to the Pt nanoparticles obtained by chemical reduction. For Pt-TiO_2__2_T the particle sizes ranged from 1 nm to 4 nm, while the mean particle size was equal to 1.5 ± 0.6 nm. Bimetallic nanoparticles Pt/Cu deposited on the surface of titanium(IV) oxide (Pt/Cu-TiO_2__C) were characterized by a size ranging from 0.9 nm to 5 nm. The average particle size was 2.2 ± 0.8 nm, while the dominant fraction were nanoparticles with sizes ranging from 1 nm to 3 nm. Based on an energy-dispersive spectroscopy (EDS) line scan, it was observed that platinum and copper form bimetallic nanoparticles with an alloy structure. Fast Fourier transform analysis confirmed the presence of platinum nanoparticles of Pt (2 0 0) and Pt (1 1 1) planes.

Bimetallic photocatalysts Ag/Pt-TiO_2__T and Ag/Pt-TiO_2__C (see Figures S3 and S4) consisted of small alloy particles up to 10 nm and bigger aggregates of a single metal. The largest aggregates (up to 100 nm) were observed for silver particles. For photocatalysts reduced thermally, silver aggregates were significantly larger in comparison to chemically reduced metals for Ag/Pt-TiO_2__C sample. Small nanoparticles consisted of silver and platinum alloys of Ag (1 1 1), Pt (2 0 0) and Pt (1 1 1) crystalline planes.

### 3.2. Photocatalytic Activity

The photocatalytic activity was evaluated in 2-propanol oxidation and hydrogen generation reactions, in order to distinguish the difference in activity in oxidation and reduction reactions, respectively.

#### 3.2.1. 2-Propanol Oxidation

**Figure 5:**
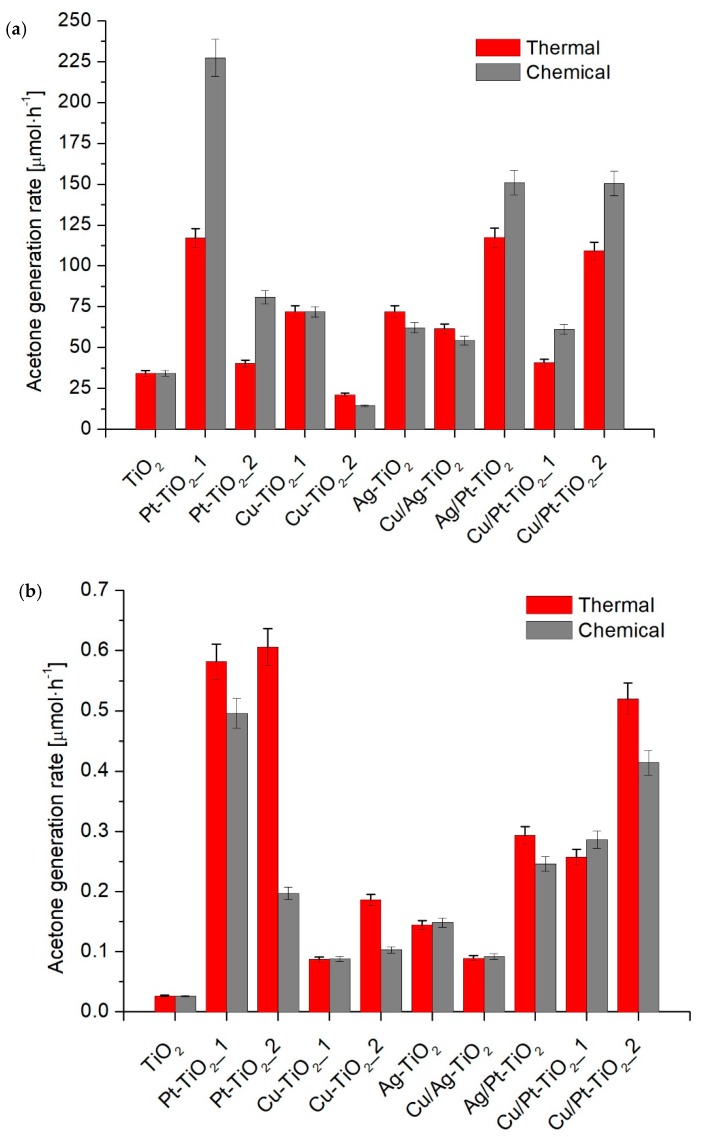
Photocatalytic activity in 2-propanol oxidation under (**a**) UV-Vis and (**b**) Vis.

Table 1 and Figure 5a,b present the results of the photocatalytic activity in 2-propanol oxidation under UV-Vis and visible light. An increase of photocatalytic activity compared to the unmodified TiO_2_ in the UV-Vis range was observed for all photocatalysts except for Cu-TiO_2__2_T and Cu-TiO_2__2_C. For photocatalysts reduced chemically, the highest activity was observed for Pt-TiO_2__1_C, for which the acetone generation rate was 227.3 ± 11.4 μmol·h^−1^. For nanocomposites obtained via thermal reduction, the highest activity was observed for Pt-TiO_2__1_T (117.1 ± 5.9 μmol·h^−1^). The particle size was a crucial parameter that affected photocatalytic activity. For photocatalysts modified with platinum Pt-TiO_2__1_C, Pt-TiO_2__2_C, Pt-TiO_2__1_T and Pt-TiO_2__2_T, a higher activity under UV-Vis was observed for nanocomposites with larger Pt particles obtained by chemical reduction. For monometallic photocatalysts modified with silver, a similar dependence was observed. Higher activity was noted for Ag-TiO_2__T with larger Ag nanoparticles in comparison to Ag-TiO_2__C. For monometallic copper modified samples, the photocatalytic activity for both chemically and thermally reduced photocatalysts was similar. However, under Vis light radiation (λ > 450 nm) higher efficiency of 2-propanol oxidation was found for thermally reduced fine Pt or Ag particles deposited on TiO_2_ surface, characterized by smaller metal nanoparticles compared to that obtained by chemical reduction, as shown in Figure 5b.

Among photocatalysts modified with bimetallic Pt-Cu and Ag-Pt nanoparticles, higher activity in reaction of 2-propanol oxidation under UV-Vis was also observed for photocatalysts obtained using a chemical reduction method. For Cu/Ag-TiO_2__C and Cu/Ag-TiO_2__T photocatalysts, a higher activity was observed for Cu/Ag-TiO_2__T. In this regard, the influence of metal nanoparticles size on photocatalytic activity is the most significant for platinum and silver NPs. Under the irradiation from the visible range (λ > 450 nm) the highest activity was observed for Pt-TiO_2__2_T (0.606 ± 0.030 μmol·h^−1^), while the lowest for TiO_2_ modified with Cu and Ag/Cu species (see samples Cu-TiO_2__1_C (0.088 μmolh^−1^), Cu-TiO_2__1_T (0.087 ± 0.004 μmol·h^−1^), Cu/Ag-TiO_2__C (0.092 ± 0.005 μmol·h^−1^) and Cu/Ag-TiO_2__T (0.089 ± 0.004 μmol·h^-1^), respectively.

Among bimetallic photocatalysts with a metal content of 0.5 mol % Cu and 0.1 mol % Pt, slightly higher activity was observed for Cu/Pt-TiO_2__1_C than for Cu/Pt-TiO_2__1_T. For Cu-TiO_2__1_C, Cu-TiO_2__1_T, Ag-TiO_2__C, Ag-TiO_2__T, Cu/Ag-TiO_2__C and Cu/Ag-TiO_2__T photocatalysts, no significant differences in photocatalytic activity were found. Moreover, the enhanced activity of Ag/Pt-TiO_2__C compared to Ag/Pt-TiO_2__T indicates the predominant role of the particle size of Pt over the size of Ag particles.

It was observed that the loading of metal nanoparticles also had an impact on photocatalytic activity. For Pt-TiO_2__2_C and Pt-TiO_2__1_C, an increment in Pt content from 0.05% to 0.1% mol TiO_2_ resulted in almost 3-times higher photooxidation of 2-propanol to acetone, whereas for Cu-TiO_2__2_C and Cu-TiO_2__1_C increasing the content from 0.1 to 0.5 mol % of TiO_2_ resulted in a five-fold increase in photocatalytic activity. For bimetallic particles modified TiO_2_ photocatalysts Cu/Pt-TiO_2__1_C, Cu/Pt-TiO_2__2_C, Cu/Pt-TiO_2__1_T and Cu/Pt-TiO_2__2_T, an increase in metal content for both chemically and thermally reduced photocatalysts resulted in almost twice the reduction of nanocomposite activity. Too high metal loading on the surface of titanium(IV) oxide can affect the photocatalytic activity as a result of hydroxylation degree reduction of the semiconductor surface and aggregation of metal particles that act as recombination centers for charge carriers [54]. Wang et al. [55] and Sun et al. [56] also observed a decrease in photocatalytic activity with an increase in metal content. They proposed that too high metal dispersion on the surface of TiO_2_ can act as an internal filter that limits the absorption of irradiation by titanium (IV) oxide.

As shown in Figure 5b and Table 1 under visible range (λ > 450 nm) a decrease in photocatalytic activity was only observed for Pt-TiO_2__1_C with higher metal content than the Pt-TiO_2__2_C sample. The acetone generation rate was equal to 0.496 ± 0.025 μmol·h^−1^ and 0.197 ± 0.010 μmol·h^−1^ for Pt-TiO_2__1_C and Pt-TiO_2__2_C, respectively.

#### 3.2.2. Hydrogen Generation

**Figure 6:**
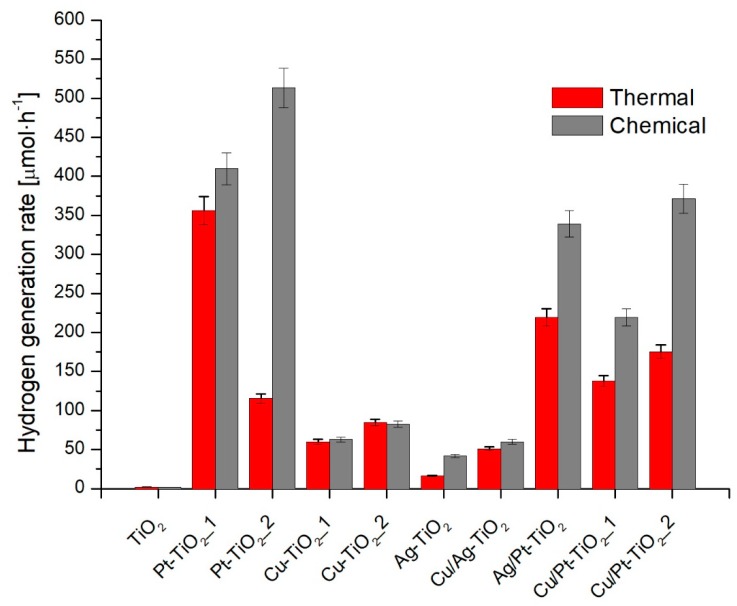
Photocatalytic activity in hydrogen generation under UV-Vis.

Photocatalytic activity in reduction reaction was evaluated in hydrogen generation reaction under UV-Vis irradiation in the presence of methanol as a hole scavenger. Results are presented in Figure 6 and in Table 1. As it was expected, the activity of bare titanium (IV) oxide was negligible. Hydrogen generation rate equaled to 1.9 ± 0.1 μmol·h^−1^. The modification of TiO_2_ surface with metal nanoparticles resulted in an increase in the activity for each of the metallic photocatalysts. The highest activities were observed for mono- and bi-metallic photocatalysts modified with platinum obtained by the chemical reduction method and characterized by larger Pt particles as compared to Pt particles obtained by thermal reduction. For Cu-TiO_2__1_C, Cu-TiO_2__2_C, Cu-TiO_2__2_C and Cu-TiO_2__2_T, there were no significant differences in photocatalytic activities between photocatalysts obtained by chemical and thermal reduction. Higher photocatalytic activity was observed for Ag and Ag/Cu-modified TiO_2_ obtained by chemical reduction, compared to thermally reduced metal ions on TiO_2_ surface. The larger silver particles were synthesized during thermal treatment rather than for chemical reduction using a strong reducing agent (NaBH_4_). For photocatalysts obtained by chemical reduction, a decrease in activity was observed with an increase in the modifier content. For monometallic photocatalysts, the highest activity was observed for TiO_2_ modified with platinum, higher than was found with copper and further with silver nanoparticles. For nanocomposites modified with bimetallic nanoparticles, no increase in photocatalytic activity was observed compared to the monometallic photocatalysts with the same metal content. The results obtained are consistent with literature reports. The results of hydrogen generation may be correlated with the metalwork function (Φ), which decreased according to dependence Φ_Pt_ > Φ_Cu_ > Φ_Ag_ [19,57].

#### 3.2.3. Action Spectra of Pt-TiO_2__1_T and TiO_2_

**Figure 7:**
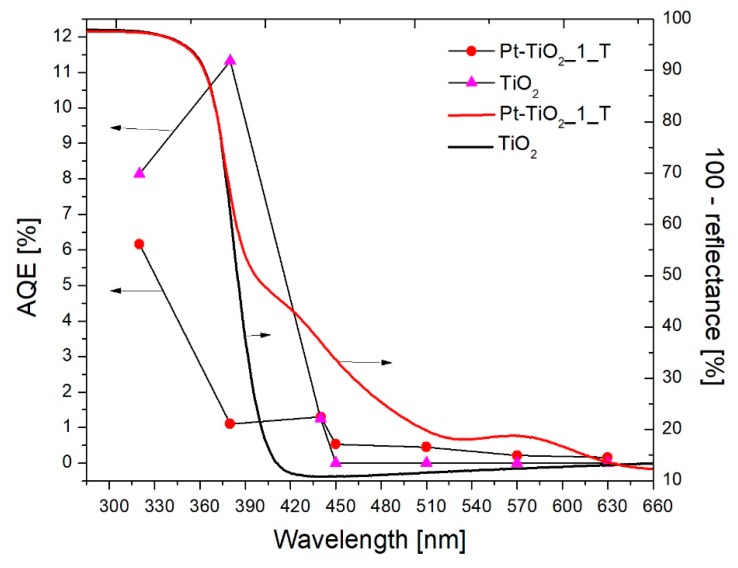
Spectral activity and absorption spectra in phenol oxidation for TiO_2_ and Pt-TiO_2__2_T. (AQE—Apparent Quantum Efficiency).

The photocatalytic activity was examined in phenol oxidation reaction under monochromatic irradiation of 320, 380, 440, 450, 510, 570 and 630 nm. Results of spectral activity measurements for TiO_2_ and Pt-TiO_2__1_T are presented in Figure 7. Apparent quantum yields of phenol decomposition were calculated in order to benzoquinone generation, as a primary reaction intermediate during phenol oxidation. In the UV region, bare titanium (IV) oxide exhibited higher activity compared to Pt-TiO_2__1_T. For unmodified titanium (IV) oxide, apparent quantum yields reached 8.14% and 11.32% under 320 nm and 380 nm, respectively, while for Pt-TiO_2__1_T 6.16% and 1.1%. Sun et al. [56] also observed the lower activity of platinum-modified photocatalysts in comparison to bare TiO_2_ during phenol oxidation under UV irradiation. They stated that lower photocatalytic activity results from decreased light absorption of TiO_2_ due to Pt NPs interfering. Water reduction to hydrogen consumes a part of photogenerated electrons, which can take part in oxygen reduction forming reactive oxygen species.

Under visible light irradiation (λ ≥ 440 nm) higher photocatalytic activity was observed for platinum-modified nanocomposite. Under λ = 450 nm bare TiO_2_ exhibited no activity, while Pt-TiO_2__1_T exhibited activity in through almost whole examined region. As the wavelength of incident light increased, the quantum yield decreased and under λ = 630 nm no activity was denoted. In the visible spectrum, the highest activity was recorded in the wavelength range from 440 to 450 nm, which confirms the existence of a platinum surface plasmon resonance.

**Figure 8:**
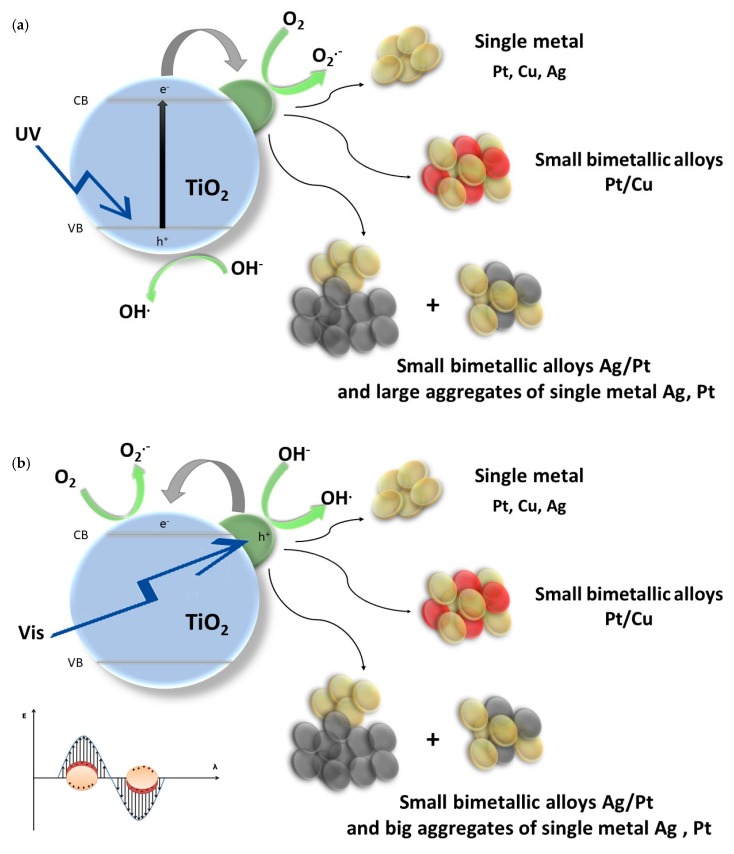
Schematic illustration of metal-TiO_2_ nanocomposite excitation under (**a**) UV and (**b**) under Vis.

Platinum nanoparticles deposited on semiconductor surface may play a different role during Pt-TiO_2_ nanocomposite excitation upon UV or visible light. The schematic illustration of photocatalytic degradation mechanism is presented in Figure 8a,b. Under UV irradiation (Figure 8a) TiO_2_ particle is the main excitation center. Photoinduced electrons from TiO_2_ valence band are transferred to the conduction band and then to the metal nanoparticle. The electron transfer results from differences in a distribution of the Fermi levels (E_f_) and generation of a Schottky barrier. When a metal work function is different than the electron affinity of the semiconductor conduction band, during the excitation of the TiO_2_ particle the excited electrons migrate towards the material of a higher value of the work function until the electrochemical equilibrium is achieved [58,59]. As a result of the Shottky barrier generation at the metal-semiconductor interface, excited electrons are trapped by a metal particle reducing the recombination of charge carriers. Under visible light, the role of an excitation center is attributed to metal nanoparticles (see Figure 8b). In the case of Pt, visible light promotes an excitation of electrons located at 5 d band and its junction to the conduction band of TiO_2_ [4,5,60,61]. Moreover, as it was previously reported by Lv et al. [62] under visible light Pt nanoparticles also act as a center of reactive oxygen species generation. The mechanism of charge carrier transport and trapping may be different when TiO_2_ is modified with other elements of compounds [63,64,65,66]. Nagakawa et al. [65,66] investigated the photocatalytic activity of CdS/WO_3_/CdWO_4_ and CdS/SiC/TiO_2_ composites modified with platinum nanoparticles in hydrogen generation reaction under visible light. The highest activity was observed for semiconductor systems modified with Pt NPs. The authors’ photocatalytic activity was attributed to the cascade carrier transfer of electrons between the semiconductors and capturing of electrons on platinum nanoparticles. Yoon et al. [64] investigated a mechanism of charge carrier transport in titanium(IV) oxide-graphene nanodots system. They stated that the electron transfer between TiO_2_ and adsorbed graphene processes through the charge transfer from HOMO (highest occupied molecular orbital) of organic adsorbate to the titanium (IV) oxide conduction band. Lee at al. [63] examined the charge transfer from adsorbed methylene blue (MB) molecule to a TiO_2_-graphene hybrid nanocomposite. They reported that high photocatalytic activity and efficient charge transfer between MB molecule and graphene-titanium (IV) oxide results from the narrowing of the TiO_2_-graphene bandgap. Narrowing of the bandgap may also lead to TiO_2_ excitation under visible light.

#### 3.2.4. Photocatalytic Activity in the Vis_LED System

Based on the quantum yield analysis of phenol oxidation, it was concluded that the photocatalytic activity of TiO_2_ modified with platinum nanoparticles corresponds to the excitation of the nanocomposite with radiation in the range from 320 to 450 nm. In this regard, the activity of the Pt-TiO_2__1_T photocatalyst was analyzed in the phenol degradation reaction under irradiation emitted by the LEDs with the emission maximum at λ = 415 nm. LED diodes may be an environmentally friendly light source with lower maintenance costs and longer operation time [67]. The phenol degradation rate constant was equal to 0.3 10^−2^ min^−1^. After 120 min of irradiation, about 30% of the phenol was oxidized. The results are shown in Figure S5. The phenol oxidation reaction proceeded linearly in the first 40 min of irradiation. After that time inhibition of the reaction was observed. Decrease of the reaction rate may result from the formation of intermediates products, including cyclohexa-2,5-diene-1,4-dione (1,4-benzoquinone). The presence of 1,4-benzoquinone in the reaction medium may significantly reduce the reaction rate due to the scavenging of superoxide anions [35,68].

#### 3.2.5. Antimicrobial Properties

**Figure 9:**
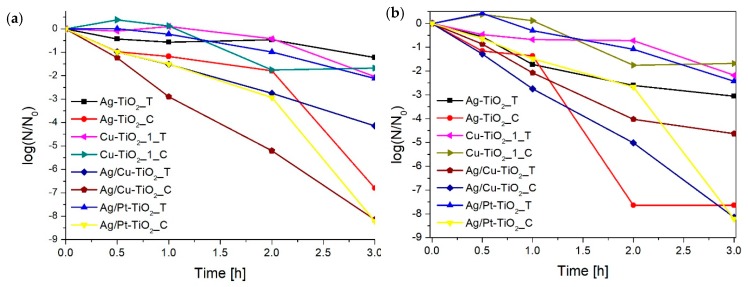
Antimicrobial activity (**a**) in the dark and (**b**) under visible light (λ > 450 nm).

The biocidal activity was tested on an *E. coli* strain in the aqueous phase. Despite the presence in the human body, *E. coli* was chosen as a model microorganism since infection with this bacterium can lead to serious health complications, such as food poisoning or urinary tract infection. Figure 9 and Table 3 present the results of biocidal activity. The tests were carried out under Vis radiation (λ > 450 nm) and in the dark. Table 3 lists the log(N/N_0_) values, where N is the number of survival bacteria, N_0_ is starting amount of bacteria, only for nanocomposites that have shown biocidal activity and for TiO_2_ as the reference material. The unmodified titanium(IV) oxide did not show antimicrobial activity. The highest activity was observed for bimetallic photocatalysts obtained by chemical reduction with small silver particles: Cu/Ag-TiO_2__C and Ag/Pt-TiO_2__C. For these nanocomposites, 100% of the bacterial cells were inactivated equally under irradiation and in the dark. In the case of other photocatalysts, the effect of radiation on the improvement of biocidal activity was observed. For the Cu/Ag-TiO_2__T nanocomposite, the efficiency of the photocatalytic disinfection was nearly twice as high when compared to the dark. It was observed that the activity of photocatalysts results mainly from differences in the size of silver particles. The smaller the silver nanoparticles, the higher biocidal activity. The Ag-TiO_2__C biocidal activity was twice as high as Ag-TiO_2__T–log(N/N_0_) values under irradiation, which were −7.63 and −3,06, respectively. A similar dependence was observed for Cu/Ag-TiO_2__C and Cu/Ag-TiO_2__T, and Ag/Pt-TiO_2__C and Ag/Pt-TiO_2__T.

## 4. Discussion and Concluding Remarks

Titanium (IV) oxide photocatalysts modified with mono- and bimetallic nanoparticles of silver, platinum and copper were obtained via thermal and chemical reduction. It was observed that the size of nanoparticles deposited on TiO_2_ surface determined the photocatalytic activity. The differences in the metal NPs size result from the reduction method used for preparation, interactions of metal with support and between deposited metal nanoparticles [26,41,69]. Based on the results of XRD, DR/UV-Vis and TEM analysis it was found that the reduction of platinum ions via the thermal method promotes the deposition of smaller nanoparticles compared to the chemical method. For Pt-TiO_2__2_C and Pt-TiO_2__2_T, the average size of Pt NPs was equal to 3.4 ± 0.9 nm and 1.5 ± 0.6 nm, respectively. The influence of the ion reduction method was also observed for silver nanoparticles. In contrast to platinum, it was found that larger Ag particles were obtained using a thermal reduction method. The average size of silver oxide crystallites for Ag/Pt-TiO_2__C and Ag/Pt-TiO_2__T was 27 nm and 57 nm, respectively. TEM analysis also confirmed that the larger silver aggregates were formed in an annealed sample. Interactions of Pt ions with TiO_2_ matrix are much stronger than of silver, therefore Pt NPs are protected against aggregation. Moreover, the binding energy between Ag ions and TiO_2_ is lower than for Pt, which results in the growth of greater Ag clusters compared to Pt [26,41]. Silver particles tend to aggregate upon the annealing process. For copper species, no dependence was observed.

Based on TEM analysis it was also observed that the size of platinum nanoparticles is also affected by the type of coupled metal. For Ag/Pt-TiO_2__C, the presence of small Ag-Pt alloy particles (5–10 nm) as well as segregation with the formation of bigger aggregates (up to 100 nm) of Pt and Ag were noticed, while for Cu/Pt-TiO_2__2_C only the formation of small Cu-Pt alloy particles with an average size of was 2.2 ± 0.8 nm was observed.

Photocatalytic activity in the 2-propanol oxidation reaction varied depending on the type of metal, the range of used irradiation and the size of the particles. Under UV-Vis irradiation, the highest activity was observed for photocatalysts containing Pt nanoparticles reduced chemically, while under visible light irradiation for photocatalysts reduced thermally. The activity of monometallic TiO_2_ nanocomposites modified with silver and copper was similar. In the literature, it can be found that the photocatalytic activity of Pt-TiO_2_ photocatalysts is very sensitive to Pt NPs [26,70]. Based on our results it can be concluded that the photoactivity of tested systems deposited on TiO_2_ depends mainly on the platinum nanoparticles size. The small sizes of metal nanoparticles enhance the photocatalytic activity due to developing of TiO_2_-metal contact surface, which promotes a more efficient charge carrier transfer between the titania and metal nanoparticles.

In the hydrogen generation reaction. a higher activity was observed for photocatalysts obtained by chemical reduction. The efficiency of hydrogen generation for monometallic nanocomposites decreased in the following order, Pt > Cu > Ag. The obtained results correlate photocatalytic activity with a work function of particular metals. The efficiency of the hydrogen generation reaction may also depend on the oxidation state of the deposited metals. Xing et al. [71] investigated the effect of the platinum oxidation state on hydrogen generation efficiency. They observed a higher activity for platinum oxides than for Pt^0^. The authors attributed this to the lower energy of PtO_2_-H_2_ adsorption than Pt-H_2_. In the literature, the synergistic effect of modification of titanium(IV) oxide with bimetallic nanoparticles, e.g., Au-Pd, Au-Pt, Cu-Pt and Cu-Au, in the hydrogen generation process is reported [72,73,74,75,76]. However, modification with bimetallic nanoparticles does not always lead to synergy in the photocatalytic reduction [77,78]. In our study, TiO_2_ nanocomposites modified with bimetallic nanoparticles showed lower or similar activity compared to that of monometallic photocatalysts. Nadeem et al. [77] investigated the photocatalytic activity of TiO_2_ modified with mono- and bimetallic nanoparticles of silver and palladium in hydrogen generation under UV light. They observed that the highest hydrogen generation rates were for a sample containing only Pd NPs. They stated that the enhanced activity of Pd-TiO_2_ over Ag-TiO_2_ and Ag/Pd-TiO_2_ photocatalysts results from the palladium high work function and thus effective electron metal to TiO_2_ CB due to a high work function. They also stated that lower activity of silver-modified photocatalysts may result from the tendency of Ag to get oxidized in aqueos conditions.

The mechanism of photocatalytic activity excitation was investigated for phenol oxidation for bare TiO_2_ and Pt-TiO_2__1_T under 320, 380, 440, 450, 510 and 570 nm. It was found that platinum-modified photocatalyst is excited by irradiation of wavelength up to 450 nm. Despite the increased absorption intensity, for Pt-TiO_2__1_T, irradiation with wavelengths above 450 nm, the apparent quantum yield of reaction did not exceed 1%. Measurements of action spectra are important information in the process of selecting the radiation source. An insight into the activity of nanocomposites under specific wavelengths allows the selection of the radiation sources with a narrower emission range, e.g., LEDs.

Experiments on *E. coli* deactivation revealed the key effect of silver particle properties on biocidal activity. The highest activity was obtained for silver-modified nanocomposites obtained by chemical reduction, characterized by small particle size. The improvement of antimicrobial properties of metal-modified titanium(IV) oxide is possible due to the synergism of the antimicrobial properties of metals and reactive oxygen species generation on the TiO_2_ surface [3,79,80,81,82,83]. For gram-negative strains, silver nanoparticles are able to absorb and accumulate on the outer cell wall leading to the disintegration of the lipid bilayer and an increase in its permeability. Moreover, silver can be embedded into the DNA, leading to the slow down of the replication process and bacteria growth inhibition as a result of the bonding with oxygen, sulphur and nitrogen atoms [84,85].

## Figures and Tables

**Table 1:** Characteristic of physicochemical properties and photocatalytic activity of as-prepared photocatalysts.

Sample Label	Metal Content Used for Preparation [% mol]			Anatase Crystallite Size [nm]	2-Propanol Photooxidation Rate [µmol·h^−1^]		Hydrogen Generation Rate [µmol·h^−1^] UV-Vis
	Pt	Ag	Cu		UV-Vis	Vis	
TiO_2_	0	0	0	12	34.3 ± 1.7	0.026 ± 0.001	1.9 ± 0.1
Pt-TiO_2__1_C	0.1	0	0	12	227.3 ± 11.4	0.496 ± 0.025	409.5 ± 20.5
Pt-TiO_2__2_C	0.05	0	0	14	80.8 ± 4.1	0.197 ± 0.010	501.4 ± 25.1
Cu-TiO_2__1_C	0	0	0.5	13	61.1 ± 3.1	0.088 ± 0.004	63.1 ± 3.2
Cu-TiO_2__2_C	0	0	0.1	15	14.3 ± 0.7	0.103 ± 0.005	81.1 ± 4.1
Ag-TiO_2__C	0	0.5	0	14	62.1 ± 3.1	0.148 ± 0.007	42.2 ± 2.1
Cu/Ag-TiO_2__C	0	0.5	0.5	12	54.3 ± 2.7	0.092 ± 0.005	60.1 ± 3.1
Ag/Pt-TiO_2__C	0.1	0.5	0	12	151.1 ± 7.6	0.246 ± 0.012	338.9 ± 16.9
Cu/Pt-TiO_2__1_C	0.1	0	0.5	14	61.2 ± 3.1	0.286 ± 0.014	219.4 ± 10.9
Cu/Pt-TiO_2__2_C	0.05	0	0.1	13	150.5 ± 7.5	0.414 ± 0.021	371.1 ± 18.5
Pt-TiO_2__1_T	0.1	0	0	13	117.1 ± 5.9	0.582 ± 0.029	355.9 ± 17.8
Pt-TiO_2__2_T	0.05	0	0	13	40.2 ± 2.1	0.606 ± 0.030	113.5 ± 5.7
Cu-TiO_2__1_T	0	0	0.5	12	71.9 ± 3.6	0.087 ± 0.004	60.1 ± 3
Cu-TiO_2__2_T	0	0	0.1	14	21.1 ± 1.1	0.186 ± 0.009	82.3 ± 4.1
Ag-TiO_2__T	0	0.5	0	12	72.1 ± 3.6	0.144 ± 0.007	16.3 ± 0.8
Cu/Ag-TiO_2__T	0	0.5	0.5	14	61.5 ± 3.1	0.089 ± 0.004	51.1 ± 2.5
Ag/Pt-TiO_2__T	0.1	0.5	0	12	117.3 ± 5.9	0.293 ± 0.014	219.1 ± 10.9
Cu/Pt-TiO_2__1_T	0.1	0	0.5	13	40.7 ± 2.1	0.257 ± 0.013	137.7 ± 6.9
Cu/Pt-TiO_2__2_T	0.05	0	0.1	12	109.1 ± 5.5	0.520 ± 0.026	175.5 ± 8.8

**Table 2:** X-ray photoelectron spectroscopy (XPS) analysis of Ti, O, C, Pt, Cu and Ag.

Sample Label	Element Content [at. %]						Particle Size [nm]
	Ti 2p	O 1s	C 1s	Pt 4f	Cu 2p	Ag 3d	
Pt-TiO_2__2_C	27.1	62.9	9.9	0.1	-	-	3.4 ± 0.9
Pt-TiO_2__2_T	24.7	63.4	11.9	n.d.	-	-	1.5 ± 0.6
Ag/Pt-TiO_2__C	26.4	62.0	11.2	0.2	-	0.2	-
Ag/Pt-TiO_2__T	26.6	60.8	12.0	0.2	-	0.4	-
Ag/Cu-TiO_2__C	23.3	62.4	13.9	-	0.1	0.3	-
Ag/Cu-TiO_2__T	25.2	60.4	13.9	-	0.2	0.3	-
Cu/Pt-TiO_2__2_C	26.9	59.4	13.6	0.1	n.d.	-	2.2 ± 0.8
Cu/Pt-TiO_2__2_T	26.9	61.7	11.4	0.1	n.d.	-	-

n.a.—not analyzed.

**Table 3:** Biocidal activity against *E. coli*.

Sample Label	log(N/N_0_)	
	Under Irradiation (λ > 450 nm)	In the Dark
**TiO_2_**	−0.27	−0.42
**Cu-TiO_2__1_C**	−2.86	−1.68
**Ag-TiO_2__C**	−7.63	−6.80
**Cu/Ag-TiO_2__C**	−8.14	−8.14
**Ag/Pt-TiO_2__C**	−8.21	−8.21
**Cu-TiO_2__1_T**	−2.17	−2.05
**Ag-TiO_2__T**	−3.06	−1.22
**Cu/Ag-TiO_2__T**	−4.64	−2.14
**Ag/Pt-TiO_2__T**	−2.43	−2.12

## Supplementary Materials

The following are available online at https://www.mdpi.com/2079-4991/9/8/1129/s1, Figure S1. Emission spectrum of a set of LED diodes, Figure S2. XRD patterns of (a) chemically reduced, (b) thermally reduced and (c) magnification in the 2θ range of 26–50° for Ag/Pt-TiO_2__C and Ag/Pt-TiO_2__T, Figure S3. (a) and (b) EDS mapping, (c) TEM image and (d) FFT analysis for Ag/Pt-TiO_2__C, Figure S4. (a) Dark-field scanning transmission (DF-STEM) microscopy and EDS mapping of (b) silver, (c) platinum and (d) titanium for Ag/Pt-TiO_2__T, Figure S5. Phenol oxidation with Pt-TiO_2__1_T using LED system.
