# Supplementary material for: Morphology, Photocatalytic and Antimicrobial Properties of TiO2 Modified with Mono- and Bimetallic Copper, Platinum and Silver Nanoparticles
Source: Nanomaterials (Basel). 2019 Aug 6;9(8):1129. doi: 10.3390/nano9081129 (PMC6722503; doi:10.3390/nano9081129)
Supplement: Supplementary file 1 [file nanomaterials-09-01129-s001.pdf]

Morphology, photocatalytic and antimicrobial properties of TiO_2 modified with mono- and bimetallic copper, platinum and silver nanoparticles

Izabela Wysocka , Ewa Kowalska, Jacek Ryl, Grzegorz Nowaczyk, Anna Zielińska-Jurek

Supplementary data

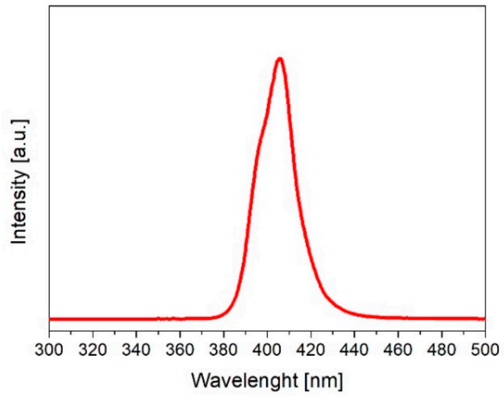

Figure S1. Emission spectrum of a set of LED diodes

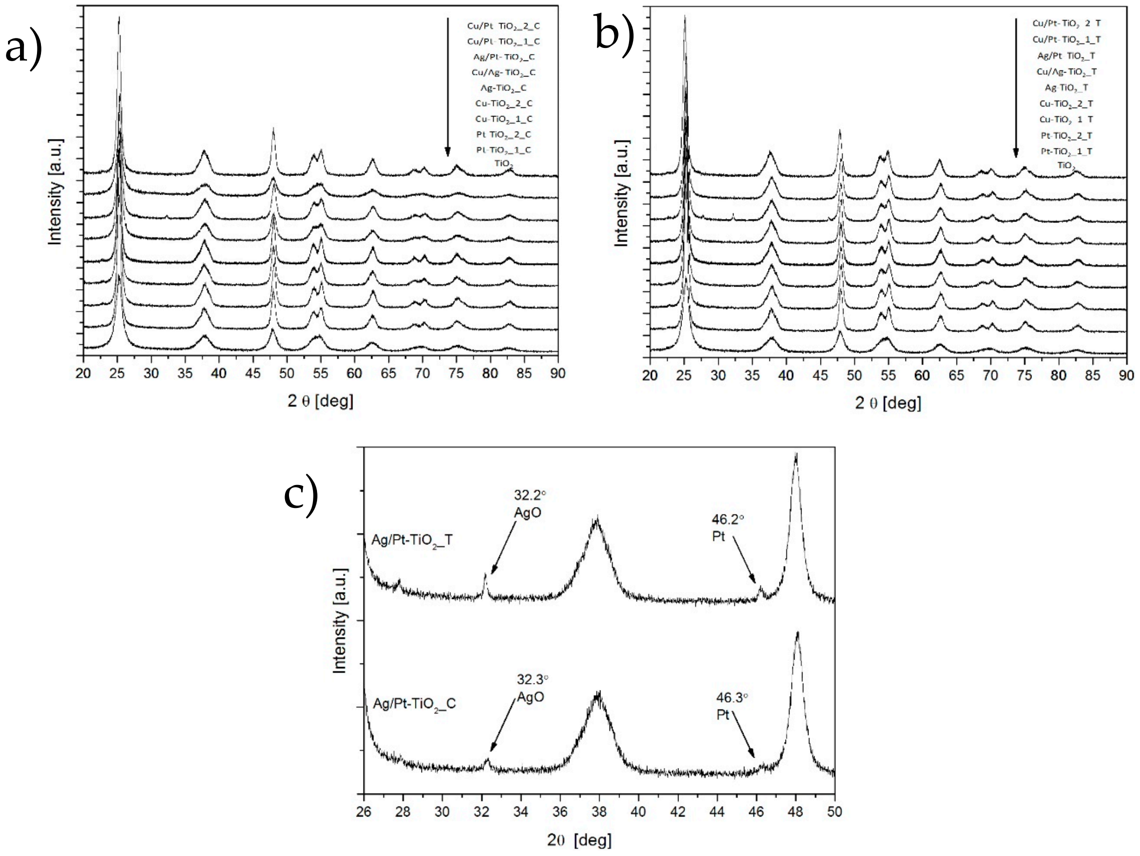

Figure S2. XRD patterns of a) chemically reduced, b) thermally reduced and c) magnification in the 2θ range of 26-50° for Ag/Pt-TiO_2_C and Ag/Pt-TiO_2_T

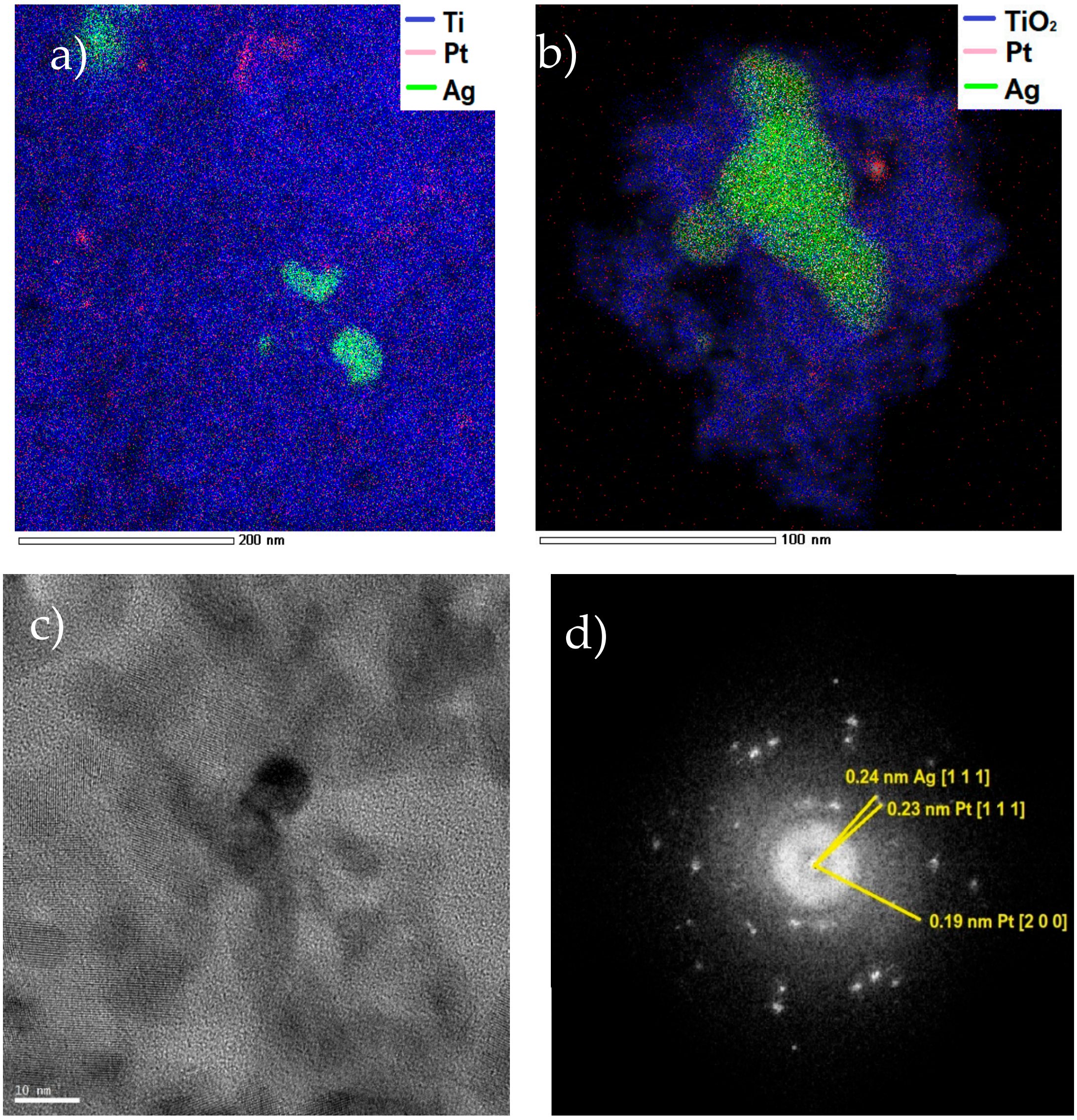

Figure S3. A) and b) EDS mapping, c) TEM image and d) FFT analysis for Ag/Pt-TiO_2_C

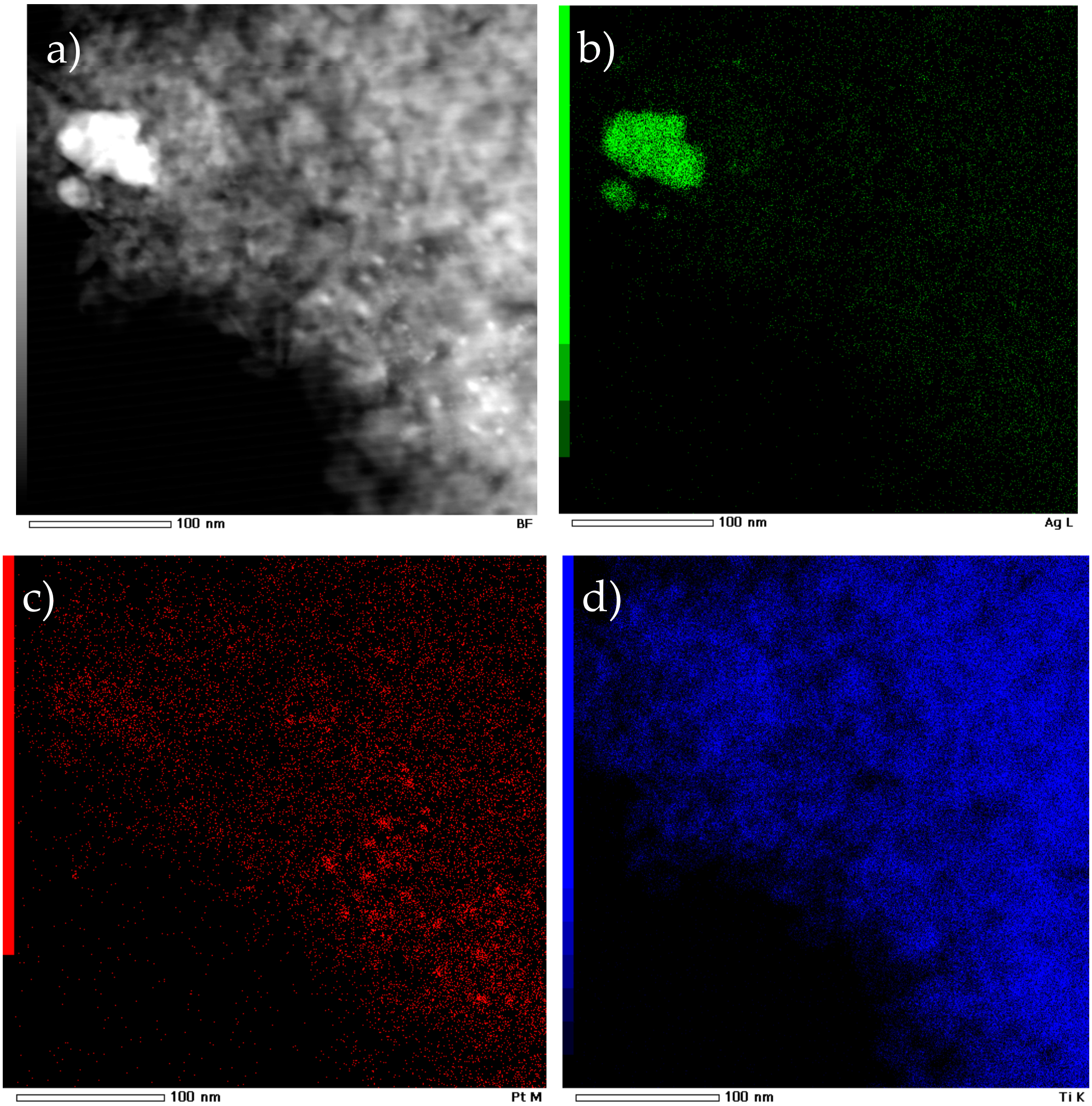

Figure S4. A) Dark-field scanning transmission (DF-STEM) microscopy and EDS mapping of b) silver, c) platinum and d) titanium for Ag/Pt-TiO_2_T

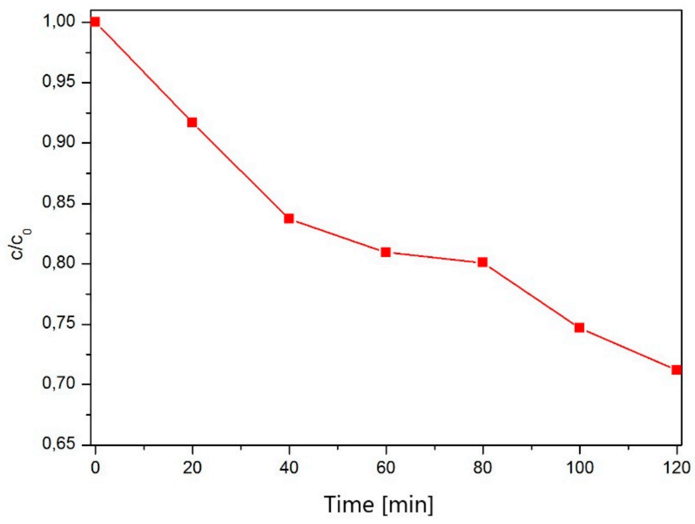

Figure S5. Phenol oxidation with Pt-TiO_2_1_T using LED system
